# Proteomics Discovery of Disease Biomarkers

**DOI:** 10.4137/bmi.s689

**Published:** 2008-06-06

**Authors:** Mamoun Ahram, Emanuel F. Petricoin

**Affiliations:** 1 Department of Pharmacology and Physiology, Faculty of Medicine, Mutah University, Mutah, Jordan; 2 Center for Applied Proteomics and Molecular Medicine, George Mason University, Virginia, U.S.A

**Keywords:** proteomics, mass spectrometry, protein microarrays, surface enhanced laser desorption/ionization, bioinformatics

## Abstract

Recent technological developments in proteomics have shown promising initiatives in identifying novel biomarkers of various diseases. Such technologies are capable of investigating multiple samples and generating large amount of data end-points. Examples of two promising proteomics technologies are mass spectrometry, including an instrument based on surface enhanced laser desorption/ionization, and protein microarrays. Proteomics data must, however, undergo analytical processing using bioinformatics. Due to limitations in proteomics tools including shortcomings in bioinformatics analysis, predictive bioinformatics can be utilized as an alternative strategy prior to performing elaborate, high-throughput proteomics procedures. This review describes mass spectrometry, protein microarrays, and bioinformatics and their roles in biomarker discovery, and highlights the significance of integration between proteomics and bioinformatics.

## Introduction

Discovery of biomarkers constitutes an essential part of biomedical research. The association of biomarkers to diseases advances understanding of cellular and molecular mechanisms of diseases since biomarkers can be direct causes of diseases, secondary players in disease initiation and progression, or mere signals of pathological conditions. In addition, the appearance of molecular biomarkers distinctly in certain pathologies will greatly assist in disease detection. More specifically, the presence of molecular biomarkers in specific stages of diseases will enable their use in disease diagnosis and prognosis. Molecular biomarkers can also play an important role in therapy as drug targets. Otherwise, such biomarkers can be used to assess the efficiency of therapeutic strategies, whereby the presence of certain molecules could be indicative of treatment goals and/or toxic effects of drugs during the course of therapy.

Research initiatives targeting biomarkers had been slow at various stages, in particular discovery and validation. Many research efforts had engaged the evaluation of very few molecules in a limited number of samples at one time. However, the initiation of molecular profiling studies aided by the completion of Human Genome Project and the recent development of biotechnological tools has revolutionized the search for disease biomarkers. Recent studies can, therefore, generate enormous amount of data end-points in a short period of time, thus termed “high-throughput.” High-throughput studies not only would enhance the discovery of novel biomarkers, but would also elucidate molecular and cellular interactions.

It has been calculated earlier that proteins constitute the main bulk of therapeutic targets accounting for more than 98% of drug targets (Drews, 2000). It has also been recently estimated that almost 10% of the genome is directly involved in pathogenesis with a fraction of these being potential targets of therapeutic intervention (Hopkins and Groom, 2002; Betz, 2005). These observations have legitimized the emergence of proteomics as a result of the biological significance of proteins. Among the many goals of proteomics is understanding all aspects of proteins including their expression, function, interaction, and structure. It is hoped that proteomics analyses lead to discovery of novel disease biomarkers that can be utilized for detection, prognostication, and treatment of diseases. In order to reach this goal, two important challenges must be encountered in terms of studying cellular proteomes, or complete sets of proteins in cells. The first is development of new technologies that would allow simultaneous investigation of numerous samples and multiple target proteins. The second challenge is the development of bioinformatics tools for the purpose of handling and analyzing the large data output.

## Role of Bioinformatics in Proteomics

With the explosion of data generated from proteomics studies, it has become a bottleneck dealing with this tremendous amount of information. Hence, the integration of bioinformatics into proteomics has become greatly needed to transform this information into meaningful knowledge. Such knowledge can be used to attain better understanding of biological systems as a whole, let alone an individual signaling pathway or a biological mechanism. A significant part of bioinformatics in proteomics, as well as other high-throughput studies, entails data management and mining. Management of proteomics data include the ability to store, search, query, and retrieve certain information; functions that have largely been achieved mainly by three major protein databases: SWISS-PROT, TrEMBL, and NCBI. These databases allow investigators access large amount of data from different species with variable redundancy and annotations in protein sequences. Nevertheless, a major shortcoming of protein databases is the existence of large amount of hypothetical and unknown proteins (Ahram and Springer, 2004).

Data mining in proteomics involves the ability to analyze and interpret the data generated by proteomics technologies. Bioinformatics tools used in proteomics can be divided into three main categories: data interpretation, pattern recognition, and predictive analysis. These tools will be discussed throughout this review. For some of these computational methods, the databases provided in SWISSS-PROT and NCBI can be integrated in order to transform proteomics data into informative output.

In this review, two proteomics technologies that have been developed with high level of innovation in order to fit into the promises of proteomics, namely mass spectrometry (MS) and protein microarrays, will be discussed. As will be illustrated, both of these technologies have been successfully used in analyzing biological and clinical samples in search for disease biomarkers. The integration of analytical bioinformatics in proteomics and their limitations will also be discussed. In addition, the growing need and development of predictive bioinformatics will be presented.

## Mass Spectrometry

Currently, mass spectrometry (MS) is the most widely used method in high-throughput proteomics studies. A mass spectrometer can measure the masses of small molecules such as peptides by converting them into ions and sorting them via a stream of electrical fields according to their mass/charge (*m*/*z*) ratio. Basic MS instruments are composed of three components: an ionization source that converts particles into ions, a mass analyzer that sorts ions according to their *m*/*z*, and an ion detector that measures *m*/*z*. Recent and continuing development and improvement of these instruments have made them greatly suitable for high-throughput proteomics studies including the invention of soft ionization methods and the innovation of hybrid instruments composed of two mass analyzers.

Two common types of soft ionization methods exist: Matrix-Assisted Laser Desorption/Ionization (MALDI) and electrospray ionization (ESI). In MALDI, samples containing peptides are embedded into specific matrix molecules. The matrix absorbs the ionization laser beam and transfers the energy into the analyte. Sample analysis by ESI, on the other hand, involves direct injection of analyte into the ionizing chamber converting peptides into smaller ions. In both approaches, ionized peptides are directed via a mass analyzer towards a detector, which generates MS spectra with each peak representing a *m*/*z* ratio of an ion.

Different mass analyzers can be combined with ESI and MALDI ionization sources. Time-of-flight (TOF) analyzer is usually associated with MALDI ion sources. In contrast, ESI can be integrated with wider variety of mass analyzers including ion trap and quadrupole. Among ion-trap mass analyzer is Fourier transform ion cyclotron resonance (FTICR), which is a special type of ion traps where ions are trapped in a magnetic field rather than an electrical one. FTICR is a powerful mass analyzer providing the highest sensitivity, resolution, and mass accuracy. For example, it has been reported that FTICR-MS can identify peptides at concentrations as low as zeptomoles (10^−21^ moles) (Belov et al. 2000).

The generated MS spectra can then be analyzed by search programs that computationally compare the actual MS spectra to hypothetical spectra. The simplest method by which proteins can be identified is via protein mass fingerprinting (PMF). This method is based on the fact that since proteins generate peptides of distinct lengths when digested by a specific protease, the identity of proteins can be determined according to their PMF. PMF works best when the analyzed sample is composed of a purified protein. Protein identification can also be performed in case of a simple mixture of proteins where database searching can be conducted repeatedly with successive removal of peptides assigned to a conclusive match (Jensen et al. 1997). A good example is the identification of protein spots in a two dimensional electrophoretic gel. Such spots commonly contain more than one protein that either possess similar molecular weight and charge (Gygi et al. 2000) or are protein contaminants such as cytokeratins (Shevchenko et al. 1996).

The introduction of tandem mass spectrometry (MS/MS) instruments has greatly improved MS technologies. These instruments are composed of two mass analyzers where following determination of peptide masses by the first mass analyzer, few peptide ions are individually selected and fragmented by collision-induced dissociation (CID) yielding even smaller ions. These ions are analyzed further by a second mass analyzer. Hybrid MS instruments include innovative combinations of mass analyzers, which can be of the same or different type. Examples include MALDI TOF-TOF where both mass analyzers are TOF, and MALDI–Qq-TOF that is composed of a quadrupole as the first mass analyzer and TOF as the second one. The dual mass analysis leads to determination of partial amino acid sequences of proteins resulting in more accurate identification of proteins than PMF only. Another major advantage of dual MS instruments is the ability to start with complex samples and the generation of amino acid sequences independently of sequence databases, although an informative database is still required for highly accurate results.

Interpretation of MS/MS data output is a rate-limiting step in accurate peptide identification. Several limitations in data analysis in accurate identification of proteins exist. In fact, Resing and Ahn (2005) have mentioned that only up to 25% of MS data could be interpreted accurately. These limitations are related to the MS instrument itself, the sample, and/or the database. MS instruments differ in their resolution and sensitivity of detection. For example, whereas ion-trap MS is of limited resolution, FTICR MS possesses the highest resolution and mass accuracy and is the most sensitive MS instrument (Domon and Aebersold, 2006). In terms of the database used for data interpretation, highly accurate results are obtained when the protein sequences in the utilized database are nearly complete. In addition, the use of large protein database can result in higher level of false-positive identifications (Resing et al. 2004; Kapp et al. 2005).

The analyzed proteins may also severely hinder accurate identification. Protein complexity may stem from their synthesis in different isoforms or their modification *in vivo* or *in vitro*. The minimal level of modification of prokaryotic proteins enables better identification than for eukaryotic proteins (Lipton et al. 2002). Amino acid differences in a protein sequence resulting from DNA polymorphism among individuals may contribute to protein complexity and, hence, prevent protein identification. Nonspecific peptides can be generated either by intracellular proteases or during sample preparation. Importantly, peptides can be chemically modified *in vitro* during sample preparation where, for example, methionines can be oxidized (Johnson et al. 2005). In fact, search programs have difficultly differentiating certain amino acids since they can carry identical or similar molecular weights such as leucine and isoleucine (Johnson et al. 2005). This is also true for phenylalanine and oxidized methionine. Pairs of amino acids may also be mistaken for a single residue such as two consecutive glycines from asparagines.

Following peptide identification by search programs, validation of these results is absolutely needed. This can be done by considering multiple factors such as missed cleavage, peptide mass, peptide modification, and number of peptides identified. Another approach is to utilize discriminant analyses such as Peptideprophet, which is a Bayesian statistical computational program that ranks peptides according to probability scores. Such a computational method enhances the performance and accuracy of search programs by reducing the rate of false-positive identifications (Kapp et al. 2005). Another discriminant approach facilitates the information provided by peptide sequences in predicting chromatographic elution time (Petritis et al. 2003). Strattmatter et al. (2004) have developed a function based on a combination of the scores generated by search programs, peptide mass, the observed versus calculated peptide mass, the cleaved nature of the peptide, and the actual versus estimated chromatographic retention time for each peptide. This method was utilized in order to identify proteins shed from the extracellular surface of hamster cells using mouse and human protein databases with limited false-positive assignments (Ahram et al. 2005).

## Surface Enhanced Laser Desorption/Ionization (SELDI)

Complex diseases cannot be labeled by a single protein biomarker. Hence, a considerable portion, if not the whole proteome, needs to be scanned in search for rather a particular profile of the particular diseases. A powerful and more versatile technology named Surface Enhanced Laser Desorption/Ionization (SELDI) branched out of MALDI-TOF mass spectrometry has been developed and can provide a solution for high-throughput analyses of cellular proteomes (Yip and Hutchens, 1992). This technology enables researchers to search for single biomarkers, a group of biomarkers, or a proteome profile. In SELDI, a sample is applied on surface of a chip rather than mixed with a matrix molecule as conducted in MALDI. The chip is then placed in a vacuum chamber of the SELDI instrument where peptides and small proteins are ionized and travel towards a detector inversely according to their masses.

With SELDI, multiple samples can be simultaneously analyzed generating numerous data points making this instrument a true high-throughput proteomics instrument. Two major advantages of SELDI is the ability to analyze highly complex samples, and the low volume of needed for analysis. The versatility of SELDI stems from the fact that chip surface can made of a defined chemical property (e.g. hydrophobic, cationic, and anionic) allowing certain classes of proteins to adsorb. Otherwise, the chip can be coated with antibodies to capture specific antigens as has been reported earlier in measuring prostate-specific antigen and prostate-specific membrane antigen (Wright et al. 2000; Xiao et al. 2001; Adam et al. 2002).

SELDI has been utilized in search for biomarkers for Alzheimer’s disease (Austen et al. 2000; Carrette et al. 2003) as well as for cancers of the prostate (Paweletz et al. 2001; Liu et al. 2003; Lehrer et al. 2005), bladder (Vlahou et al. 2001), colon (Engwegen et al. 2006), and breast (Ricolleau et al. 2006). Although direct determination of proteins represented as mass peaks is not possible, different means can be utilized to reveal the identity of specific peaks. In a recent report, SELDI analysis of cerebrospinal fluid (CSF) samples of patients with multiple sclerosis revealed the presence of a differential peak when compared to subjects with other diseases (Irani et al. 2006). This peak was identified by further MS analyses as cystatin C, an inhibitor of the lysosoaml cysteine protease cathepsin B. Although burdensome and elaborate, proteins represented by specific SELDI spectra peaks can also be identified by a series of liquid chromatography fractionation as has been illustrated by Diamond et al. (2003), Sanchez et al. (2004), and Yang et al. (2004).

In one study, proteins extracted from LCM-microdissected prostate normal and tumor cells were analyzed by SELDI. The mass spectra patterns of the proteins revealed several remarkable alterations as compared to those of matched normal samples (Petricoin et al. 2002). However, due to the dynamic heterogeneity of proteomes even within the same individual, consistent detection of differential peaks is not always feasible. This complexity has prompted the group of Petricoin and Liotta to integrate an artificial neural network algorithm to search for “hidden” patterns. In a prominent study, the group has been able to differentiate ovarian cancer patients from normal subjects and patients with other ovarian diseases with unprecedented sensitivity of 100% and specificity of 95% (Petricoin et al. 2002). This very similar approach has been utilized in detecting gastric cancers with high sensitivity and specificity in differentiating the disease (Ebert et al. 2004).

Although these results are promising, serious concerns have been raised in terms of the robustness and reproducibility of the approach. One such concern is the inability of the current SELDI-TOF instruments to directly sequence and identify the peptides/proteins that generate the discriminatory peaks, and thus be able to independently validate the markers by other analytical approaches. Some investigators suggest that these differential low-molecular-weight (LMW) products may not be produced by the diseased cells themselves, but rather they may be generated by epiphenomena within the microenvironment (Diamandis, 2003; Diamandis, 2004; Seibert et al. 2005; Poon, 2007). However, recent reports (Lowenthal et al. 2005; Lopez et al. 2007) indicate that the LMW fragments constitute unique disease-specific protein fragment isoforms that appear to emanate from low abundant tumor cell input. As we transition from patterns of unknown analytes to fingerprints of multiplexed known markers, there is agreement of the importance of quality control, quality assurance, and the development of high operating standards in order to minimize potential bias that can result from sample collection, handling and processing (Liotta and Petricoin, 2008).

## Protein Microarrays

The success of DNA microarrays has encouraged scientists to invent a similar technology for proteins, hence termed protein microarrays. Different types of protein microarrays have been introduced and can be categorized according to their end-point purpose. Similar to SELDI, protein microarrays can aid in search for single biomarkers, a group of biomarkers, or a proteome profile, depending on the type of microarray. In general, they can be categorized into three groups: expression-based, function-based, and interaction-based microarrays. Expression-based protein microarrays are more common and better developed. It aims to investigate protein expression within a sample. Expression-based microarrays can also be of two types: forward phase and reverse phase microarrays. Forward phase microarrays entail spotting thousands of bait molecules on a glass or membrane-coated slide. Each spot would then represent a specific bait for a single protein. Usually the bait molecule is an antibody (Haab, 2001), although other capture molecules such as aptamers (small DNA or RNA molecules) or phage lysates have been reported (Choi et al. 2005). By incubating a sample containing mixed populations of proteins onto the spotted slides, protein molecules would bind specifically to the corresponding bait molecule. Captured proteins can then be detected by directly labeling the proteins before applying them onto the slide. This direct labeling method has been utilized in identifying biomarkers of prostate cancer (Miller et al. 2003) and radiation-regulated proteins (Sreekumar et al. 2001). With the direct labeling method, it is possible to perform comparative expression analysis of two or more samples with proteins in each sample labeled with a distinct tag (Haab, 2005).

Otherwise, an indirect labeling method, also known as sandwich immunoassay, can be used where bound proteins are targeted by a second bait such as a different antibody that targets a different domain. This method is limited to analyzing the expression of proteins within a single sample. Although the requirement for two independent bait molecules may limit multiplexing, this method can be more specific and sensitive than the direct labeling approach since two bait molecules are required to target the same proteins rather than one molecule. The sandwich immunoassay has been illustrated in measuring the expression the epidermal growth factor receptor and ERB2 and monitoring EGF-dependent phosphorylation in human tumor cells (Nielsen et al. 2003).

Forward-phase protein microarrays are in contrast to another design of expression microarray technology termed “reverse-phase protein microarrays” (Paweletz et al. 2001). This technology involves spotting the analytes (i.e. protein extracts) rather than bait molecules, with each spot representing a single test sample. Since proteins are expressed at a wide range of orders of magnitude, lysates can be spotted at different dilutions providing an internal standard curve and an opportunity for quantitative measurement. It is worthy to mention that spotted lysates can be obtained from microdissected cells from tissues allowing for studies of pure cell populations. For example, differential protein expression in microdissected prostate cancer cells has been compared to that in patient-matched normal and premalignant cells from the same tissue samples (Paweletz et al. 2001). In addition to expression pattern, signal transduction circuitry can be studied using reverse-phase protein microarrays (Paweletz et al. 2001; Nishizuka et al. 2003; Petricoin and Liotta, 2003). Two reports have shown that the activation of Akt pathway is responsible for cell viability in both ovarian and prostate cancers as cancer cells progress from premalignancy to malignancy (Paweletz et al. 2001; Wulfkuhle et al. 2003). Whereas with forward-phase proteins microarrays, one can analyze multiple proteins in a few samples per one microarray, reverse-phase protein microarrays enable investigation of one or various proteins in multiple samples per one microarray.

Both forward-phase and reverse-phase protein microarrays are hampered, though, by the availability of a specific bait molecule. A huge endeavor of the HUPO Antibody Initiative has been undertaken by the scientists of the Swedish Royal Institute of Technology led by Mattihas Uhlén to generate, validate, catalog, and annotate antibodies that target human proteins with high specificity and low cross-reactivity to other proteins (Uhlén et al. 2005). The main objective of this initiative is to create a protein atlas for localized exression of proteins in human tissues. Recently, the National Institutes of Health have established a similar initiative termed Protein Capture Tools that emphasizes on monoclonal antibodies (Haab et al. 2006; Hober and Uhlén, 2008). The European Union has also funded another program, Proteome Binder, with special focus not only antibodies and antibody related reagents like single chain antibodies (scFv), but also on other affinity reagents such as nucleic acid aptamers, protein scaffolds, peptides and chemical entities (Taussig et al. 2007).

The second type of protein microarrays, functional microarrays, aims to assign function to proteins. This can be achieved by conducting a micro-scale enzyme assay whereby product formation can be measured. The enzymatic activities of purified 119 yeast proteins predicted to be tyrosine kinases have been investigated using a functional microarray where kinase substrates are arrayed on a solid surface (Zhu H et al. 2000). Purified proteins are then added individually to the microarrays in the presence of ^32^Pγ-ATP. The ability of 27 of the 119 proteins to phosphorylate certain substrates has confirmed their kinase activity.

Interaction protein microarrays can play an important role in determining protein function as well in therapeutics. Interaction arrays can be designed to investigate interaction of certain proteins to various types of molecules including other proteins, peptides, nucleic acids, lipids, carbohydrates, and small molecules (MacBeath and Schreiber, 2000; Ge et al. 2000; Iyer et al. 2001; Linnell et al. 2001; Zhu et al. 2001). For example, by attaching over 90% of yeast proteins onto a microarray, Zhu and colleagues were able to identify protein-phospholipid interactions as well as new calmodulin-interacting proteins (Zhu et al. 2001). In addition, effects of DNA mutations and polymorphism on DNA-protein binding have been studied using interaction protein microarrays (Boutell et al. 2004). Both functional arrays and interaction arrays can also prove invaluable in drug discovery where binding of small ligands to protein targets can be multiplexed, in addition to analyzing the effect of different small molecules on enzyme activity.

## Predictive Bioinformatics in Proteomics

As mentioned earlier, bioinformatics is expected to play a major role in analyzing proteomics data. However, it is still a long shot to understand what these data mean and how they can be useful. A major reason for functional deficiency is the existence of large number of unidentified protein sequences in protein databases accounting for almost 60% in one report (Ahram and Springer, 2004). A branch of bioinformatics that can play an informative role in proteomics is based on predicative computational tools. Predictive bioinformatics can overcome the technical limitations of proteomics by contributing to the annotation of proteins and determination of their function and structure. Several tools have been reported by which protein localization, function and structure can be examined theoretically before moving into the experimental arena. Identification of homologous regions of proteins can lead to predicting protein function and localization. In addition, protein localization can be predicted based on presence of specific amino acid sequence. Many of these tools are offered in the SWISS-PROT and some of them are reviewed elsewhere (Emanuelsson and von Heijne, 2001).

Understanding protein topology is critical for determining protein structure and function and, hence, developing novel therapeutics. Although membrane proteins are thought to constitute 20–30% of annotated genomes (Steven and Arkin, 2000; Wallin and von Heijne, 1998; Krogh et al. 2001), the 3D structures of only 1% of these proteins have been determined (Melen et al. 2003). A class of proteins of special interest is membrane proteins, in particular plasma membrane proteins. A significant role of these proteins is that they constitute more than 45% of current drug targets (Drews, 2000) with 25%–30% of drugs targeting G-protein coupled receptors (Hopkins and Groom, 2002). Thus, construction of computational tools that predict protein topology is imperative for large-scale proteomics studies. These tools operate by predicting the presence of transmembrane segments. However, a major pitfall of all methods is the erroneous prediction of the N-terminal signal peptides as transmembrane segments as a result of their hydrophobic nature (Ahram and Springer, 2004).

In a recent study, a human proteome database was analyzed using five predictive computational methods in search for membrane proteins (Ahram et al. 2006). These five methods are commonly used and based on different computational approaches. In order to eliminate false-positive predictions, a sixth method, SignalP, which discriminates between signal peptides and transmembrane segments, have also been utilized. Based on these analyses, the ratio of human proteins with transmembrane segments is estimated to fall between 15% and 39% with a consensus of 13%. Such a broad range of prediction depends on the selectivity of the individual method in predicting integral membrane proteins. These methods can play a critical role in determining protein structure and, hence, identifying suitable drug targets in humans.

Another major effort was conducted in search for novel secreted proteins using both biological approach and a computational strategy (Clark et al. 2003). The latter was based on analysis of genomic and expressed sequence tags (ESTs). This study resulted in the isolation of over 1000 cDNA clones with 25% of them representing novel genes. An important conclusion of this study is the significance of applying multi-directed approaches in the identification of proteins.

## Conclusion

Exciting advances have been posted in proteomics. These advances have mainly been observed in the technical field with the development and improvement of technologies such as mass spectrometry, SELDI, and protein microarrays. However, these technologies are still limited in many areas including specificity and sensitivity of detection. In addition, data mining can also see significant attention for better accuracy of protein analysis. It is the integration of these technologies as well as the development of bioinformatics tools that can speed up the discovery of protein targets for therapeutics and lead to more accurate and safer drugs.
